# A Narrative Review of the Potential Injuries in Coastal Rowing Beach Sprint: An Approach to Improve Medical Care in Training and Competition

**DOI:** 10.7759/cureus.101068

**Published:** 2026-01-08

**Authors:** Paul Hans Richard Martin, Georg Gosheger, Kristian Nikolaus Schneider

**Affiliations:** 1 Department of Orthopaedics and Tumour Orthopaedics, Münster University Hospital - Albert-Schweitzer Campus 1, Münster, DEU

**Keywords:** coastal rowing beach sprint, injury, olympic games, olympics, prevention, rowing

## Abstract

Coastal rowing beach sprint is scheduled for inclusion in the Olympic program at the Los Angeles 2028 Games. This discipline represents a modification of traditional Olympic rowing, featuring head-to-head competition that combines short beach sprints with an offshore rowing course around a buoy slalom before returning to shore. Compared with classic Olympic rowing, Beach Sprint rowing presents a distinct injury profile, with the potential to exacerbate injury patterns already common in rowing while introducing additional environmental hazards. Factors such as larger waves, stronger currents, and ocean swell may increase the incidence of acute traumatic injuries in a sport traditionally dominated by overuse injuries. Consequently, team physicians and medical personnel should be familiar with the specific risks and injury patterns associated with this discipline, ensure that appropriate medical equipment is readily available near the course, and establish efficient procedures for safe and rapid evacuation of athletes from the beach. Additionally, the provision of adequate shade, access to fresh water for cleansing potential pathogen entry sites, and appropriate protective equipment in conditions of high waves or strong winds are important measures to reduce the risk of injury and infection.

## Introduction and background

The relatively recently developed discipline of coastal rowing beach sprint will be Olympic for the first time at the 2028 Olympic Games in Los Angeles, USA [1]. Despite its rapidly growing popularity and inclusion in the Olympic program, scientific evidence regarding discipline-specific risks, injury patterns, and preventive strategies remains limited.

This lack of data poses challenges for medical personnel and coaches in adequately preparing for the demands of this event. A comprehensive understanding of the sport’s risk factors, injury mechanisms, and underlying biomechanics is therefore essential to optimize injury prevention and medical management.

This narrative review aims to identify potential injury risk factors associated with coastal rowing beach sprint and to explore preventive measures, thereby providing a practical framework to improve medical care for athletes participating in this discipline. In addition, this review seeks to support event organizers in planning and delivering appropriate medical coverage for coastal rowing beach sprint competitors.

**Figure 1:**
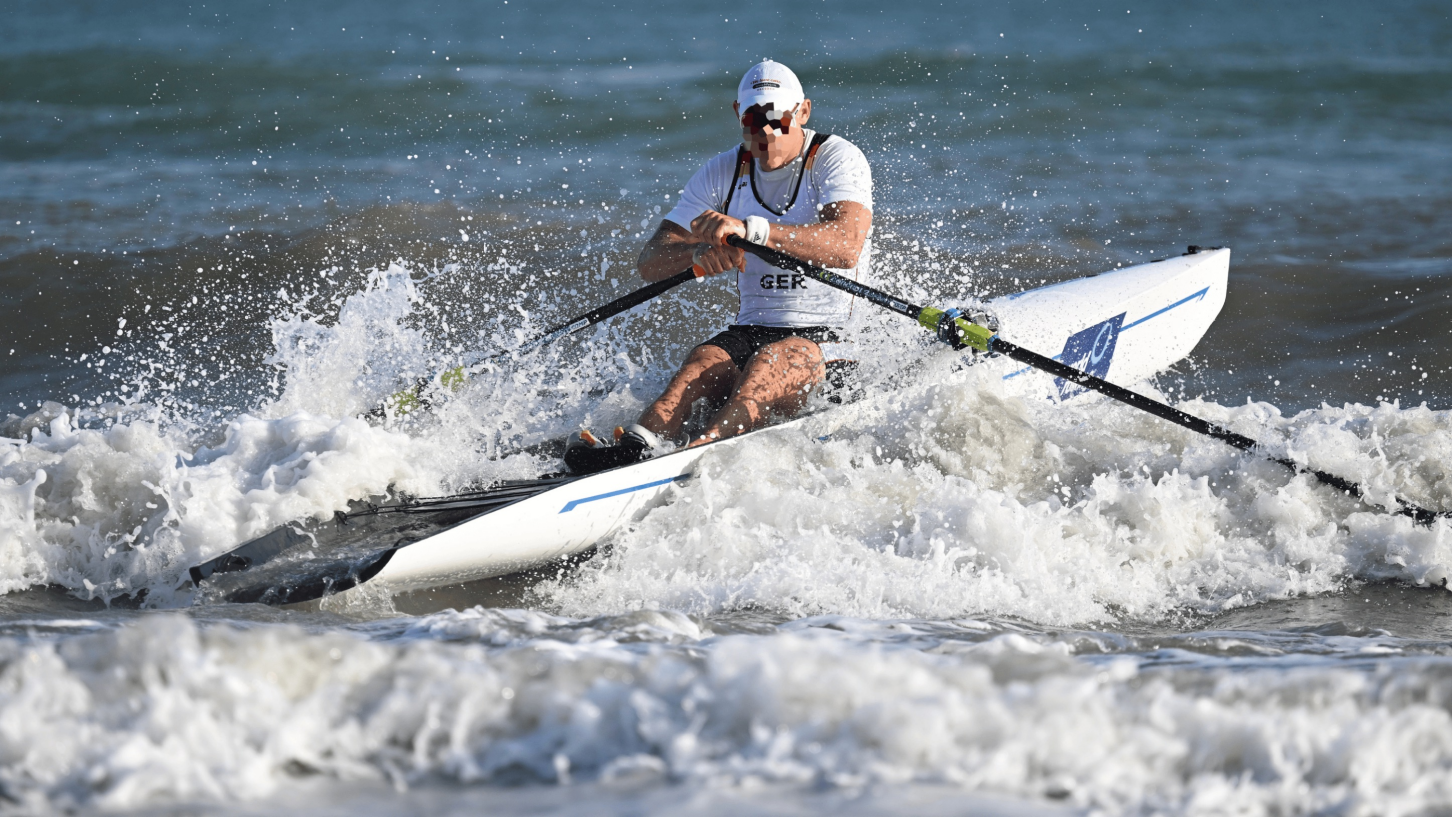
Single sculler participating at the world championships of coastal rowing beach sprint in Barletta/Italy in 2023

Coastal rowing beach sprint comprises both a running (“Beach Sprint”) and a rowing component. The race begins with a beach sprint of around 30 meters using a “Le Mans“-start style, followed by a rowing slalom around a buoy and back of around 500 meters, and concludes with a final 30-meter beach sprint to the finish line, where athletes must press a buzzer to complete the race [2,3]. The start of such a race can be seen in Figure 1. This discipline thus integrates elements of traditional Olympic rowing, surfboat rowing, and beach running, combining movement sequences in a manner reminiscent of multisport disciplines such as triathlon [4,5].

Materials and methods

Research for this narrative review was carried out using the three main academic search engines, PubMed, Google Scholar, and Science Direct. Respective search terms used were as follows: “rowing injuries”, “rowing”, “injuries”, “canoeing”, “kayaking”, “beach”, “beach volleyball”, “triathlon”, “water borne infections”, “upper extremity injuries”, “lower extremity injuries”, “seashells”, “shoulder injuries”, “spine injuries”, “wrist injuries”, “Olympic games”, “ophthalmic injuries”, “saltwater”, “dehydration”, “athletes”, “sun exposure” and “cumulative load”. To specify results, the operators AND and OR, as well as the specifications (TI) and (TIAB), were used. All results as of June 2025 were included.

Matching peer-reviewed publications as of June 2025 in English, as well as German, were screened by the first author to assess the relevance for this narrative review. Only studies discussing potential injuries and their origin, incidence, and risk factors in coastal rowing beach sprint and classic Olympic rowing or similar sports were included. Studies focusing on biomechanics, outcomes, or any other aspects not linked to the above were not included. Review articles were preferred but, given the limited number of publications, could not be used exclusively.

In addition, official documents of the World Rowing Organization (FISA) and the German Rowing Association (DRV) were screened. Furthermore, expert interviews with coaches, team doctors, and physiotherapists of the German Coastal Rowing Beach Sprint national team were performed. No quantitative data synthesis was performed, nor was it intended.

## Review

Injuries in classic Olympic rowing

In classic Olympic rowing, the incidence of injury during competition is relatively low; however, injuries are considerably more common during training, especially when contrasted with sports that have a similar training-to-competition distribution across the year. In elite-level classic Olympic rowing, the majority of injuries are minor and of low severity. These injuries are usually non-traumatic and are frequently linked to high training volumes, particularly during the transition period between winter and summer training [6-9]. Furthermore, nearly half of all injuries in classic Olympic rowing occur during land-based training, most commonly during rowing ergometer use or weight training [10].

The Rowing Motion

The rowing motion is cyclic and consists of two phases: The drive and the recovery [10]. The athlete sits in the rowing boat facing the stern, effectively going backwards in order to propel the boat forward. The drive starts with the arms fully extended while the hip, legs, and core are in full flexion, and the rowing blades are squared (turned perpendicular to the surface of the water) and fully set into the water. At this point, the body of the rower is at the most sternward position during the stroke. From this point on, the rower extends his legs, followed by his hips and glutes, before finishing with the extension of his core muscles to create the backswing, which is accompanied by a flexion and backward abduction of the arms to finish the drive. As a result, the rower has moved his whole body towards the bow of the boat as he sits on a sliding seat with only his feet firmly connected to the rowing shell, thus enabling him to shift his mass during the stroke.

This is followed by the recovery, which starts with lifting the blades out of the water and turning them parallel to the surface of the water (so-called feathering) [10]. Next, the arms are extended in front of the body; straight after that core and hips flex along with the legs, moving the body towards the stern of the boat; squaring (turning the blade perpendicular to the water); and setting the blades marks the end of the recovery [10].

Both phases together form one rowing stroke, with the recovery making up about two-thirds, while the drive only makes up one-third, timewise. Even though the general motion is the same in Coastal Rowing and classic Olympic rowing, there are major differences between these two disciplines regarding almost every other aspect of the sport, including very different demands on the athletes' training [11]. Furthermore, the unique structure of coastal rowing beach sprint hints towards a different and potentially unique risk profile due to rougher conditions while rowing at the beach.

Differences Between Coastal Rowing Beach Sprint and Classic Olympic Rowing

The most obvious difference between these two disciplines of rowing is the type of water on which the sport is performed (Table 1). Even though some Olympic rowing competitions have been held on saltwater courses (e.g. the Olympic Games in 2016 Rio/Brazil or the 2021 World Cup III in Sabaudia/Italy), Coastal Rowing events are exclusively performed at the beach.

**Table 1:** Comparison between coastal rowing beach sprint and classic Olympic rowing

Coastal rowing beach sprint	Classic Olympic rowing
30m Running, 500m row,30m running	2000m row
Competitions at the beach	Competitions on lakes, rivers etc.
Only sculling boats	Sculling and sweep rowing
Wide, short and heavy boats	Narrow, long and light boats
Mixed sex crews at the Olympics	Only same sex crews at the Olympics
Slalom course	Straight course

**Figure 2:**
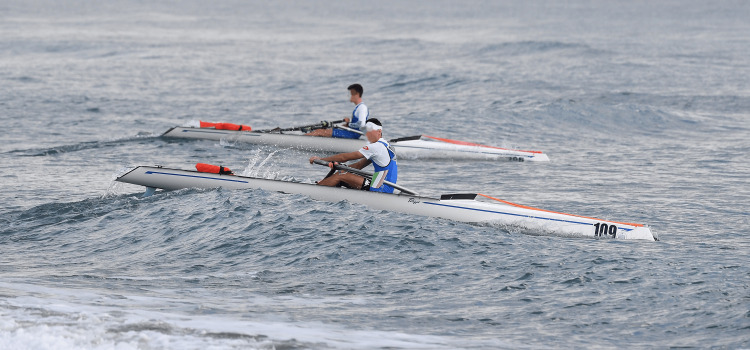
Wavey conditions during side-to-side racing in Donoratico, Italy, during the 2020 European Coastal Challenge

The ocean as a regatta venue itself brings along several possible sources for injury and/or infection, such as waterborne disease, but most importantly, swell and waves, which can get quite big, as seen in Figure 2 [12].

Another major difference is the material being used and the boat classes. All coastal events are rowed in so-called sculling boats in which each rower holds two oars (one in each hand), enabling a single rower to move a boat all by himself (single scull or 1x). Classic Olympic rowing, on the other hand, also includes sweeping boat classes with only one oar per rower.

**Figure 3:**
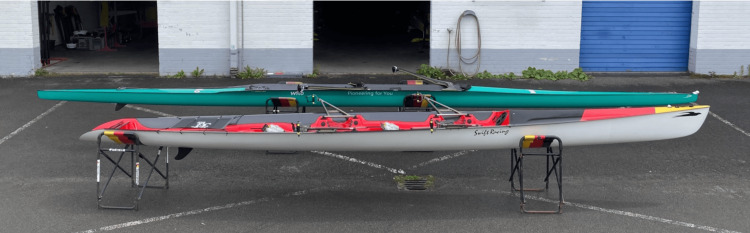
Comparison of a coastal rowing boat (front) with an elite-level classic Olympic rowing boat (back)

Even though all boats are made of carbon fiber, Kevlar, and similarly robust and lightweight materials, they differ tremendously in their weight and shape [13]. While classic rowing boats are quite narrow, long, and light, Coastal Rowing boats are built very wide and short and are heavier [14,15]. The differences are visible in Figure 3, where a classic Olympic pair in green is compared to a Coastal Rowing Double.

Every coastal rowing beach sprint regatta includes two short sprints on the beach before and after the actual rowing part of the regatta [3]. This is another difference compared to classic Olympic rowing and might also be a major risk factor regarding injuries in competition and training. Coastal rowing beach sprint competitions also include a different array of boat classes compared to classic Olympic rowing competitions. While there are only same sex crews in Olympic rowing races, coastal rowing beach sprint also includes so-called mixed doubles in which a man and woman compete together. In total, there are 10 boat classes in coastal rowing beach sprint (men's, women's, and junior women's and men's single scull, junior men's, and women's double, as well as a junior and open mixed double, an inclusion mixed double, and a coxed quadruple scull). Classic Olympic rowing, on the other hand, has 12 boat classes as it includes bigger boats reaching up to an eight, as well as sweeping boat classes (eight, coxless four, and pair).

Potential risks and injuries

Coastal rowing beach sprint and classic Olympic rowing share certain similarities, yet they represent two distinctly different disciplines. In the following section, potential risks and injuries are described according to anatomical region. An important factor influencing risk in coastal rowing beach sprint is the dense race schedule, with up to six races conducted within an eight-hour period. This competition format may increase the overall risk of injury due to cumulative physical exertion [16,17].

Injuries of the Head and Neck

Traumatic head injuries are rare in classic Olympic rowing races; however, they can still occur due to collisions, particularly during training sessions or during launching/loading procedures [18]. The same can be said for neck injuries, though overuse injuries are a little more common and mainly the result of technical flaws in the athlete's rowing technique.

Coastal rowing beach sprint athletes are most likely prone to experience similar overuse injuries, especially since waves and swell will increase the difficulty of rowing conditions while placing the rower under more stress than athletes in classic Olympic rowing experience.

In ocean kayaking, head injuries are among the four most common types of injuries, making up about 10% of all injuries reported in the sport [19]. Although kayaking is a different sport, especially regarding the main muscle groups used, compared to coastal rowing beach sprint, the similar environment suggests an increased risk for head injuries in coastal rowing beach sprint compared to classic Olympic rowing. This risk is further enhanced by contact with the boat or oars, which will be highlighted further on in the text. A study on the incidence of injuries in coastal rowing beach sprint rowing during the 2022 world championships mentions one athlete who sustained a concussion while training at the venue, which further underlines the increased possible risk for head injuries compared to classic Olympic rowing [20].

In addition to orthopaedic injuries to the head and neck, saltwater can also lead to head injuries, mostly to the eyes. The results are usually mild aberrations and a slightly blurry vision, which may resolve after a couple of days. If the water is contaminated with various pathogens, the irritation and potential infection take longer to heal and may even require ophthalmological treatment [21].

Injuries of the Spine and Trunk

Back injuries, especially those regarding the lower back and lumbar spine, are the most common injuries in classic Olympic rowing [10,22]. Resulting injuries range from non-specific low back pain to inflammation, bulging, and herniation of intervertebral discs, especially in the lumbar spine [10]. Similar incidents can be observed for white water paddling and kayaking, where it is also among the most common locations of injury [12,19].

**Figure 4:**
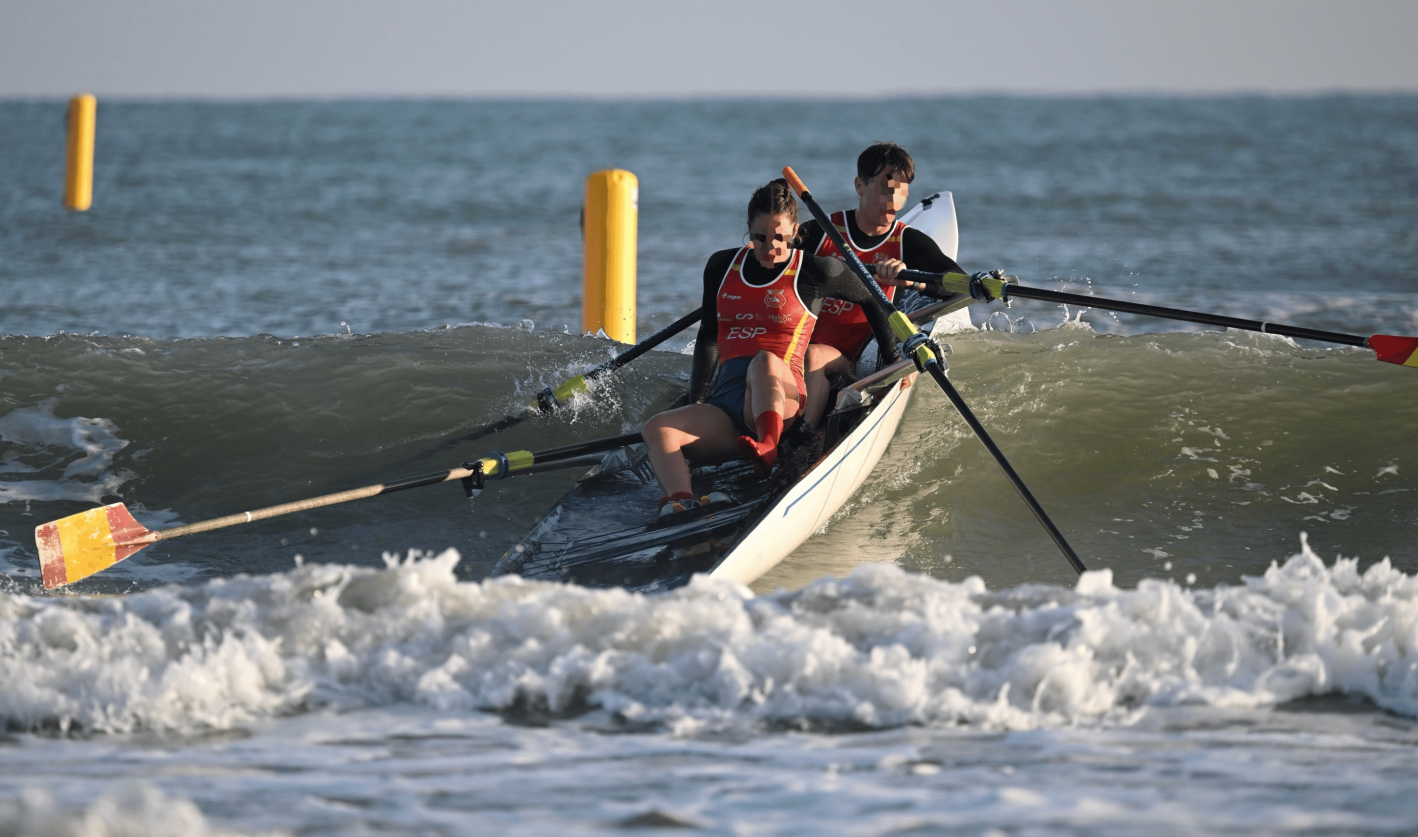
Six waves hitting a coastal double at the 2023 World Rowing Beach Sprint Finals 2023 in Barletta, Italy This image highlights the impact waves and swell have on athletes’ bodies.

**Figure 5:**
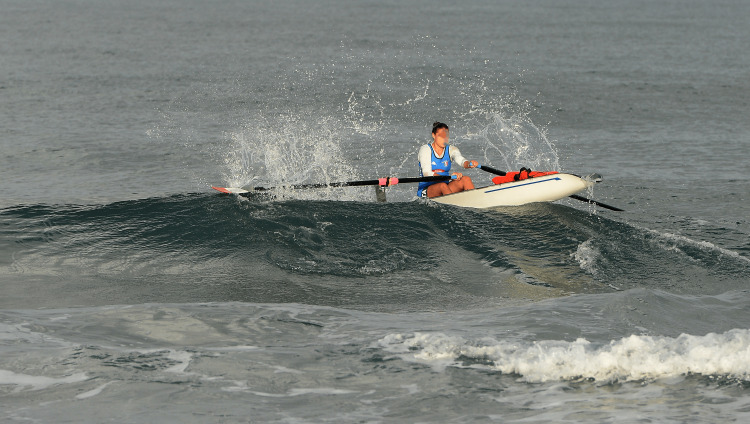
Women’s single rowing over a big wave at the 2020 European Coastal Challenge in Donoratico, Italy This image underlines the size and impact waves have on rowers during the race.

In contrast to classic Olympic rowing, where a lot of spine and trunk injuries are the result of high training volume, kayaking and white-water paddling injuries are usually traumatic (white water paddling) or due to rotational loads placed on the spine in environments with waves and swell [12,19]. Although white water rafting is not the same as coastal rowing beach sprint, there are some similarities, especially regarding the water environment (waves, swell, etc.). These similarities also hint towards a potentially increased occurrence of traumatic spine and trunk injuries compared to classic Olympic rowing, in addition to the back injuries obtained during standard endurance training while rowing on calmer waters or on the rowing machine [10,12,19]. The images presented in Figures 4-5 demonstrate the sizes of waves that coastal rowing beach sprint athletes can encounter.

Another common injury of the trunk is a stress injury of the ribs, which affects around 9% of rowers [23]. The epidemiology of these injuries has not yet been determined, but multiple factors have been brought up, such as muscular rib cage contractions, force transmission of the oar handle to the ribs, or forces of the serratus anterior and external oblique muscles during the stroke [24,25]. As a result, continuous contractions with high force are a risk factor, which means rib stress fractures are potentially also very likely to occur in coastal rowers, especially as the short distance of about 500 meters encourages stronger and faster strokes [10].

Injuries of the Upper Extremity

Most injuries of the upper extremity in classic Olympic rowing are associated with bad rowing form, overuse, or difficult conditions, which in turn lead to a decrease in rowing technique quality, thus increasing the risk for injuries even more [10,19,26]. The coastal rowing beach sprint setting most likely leads to an exaggeration of these injury potentials while bringing along new injury risks as well.

Injuries of the Shoulder

**Figure 6:**
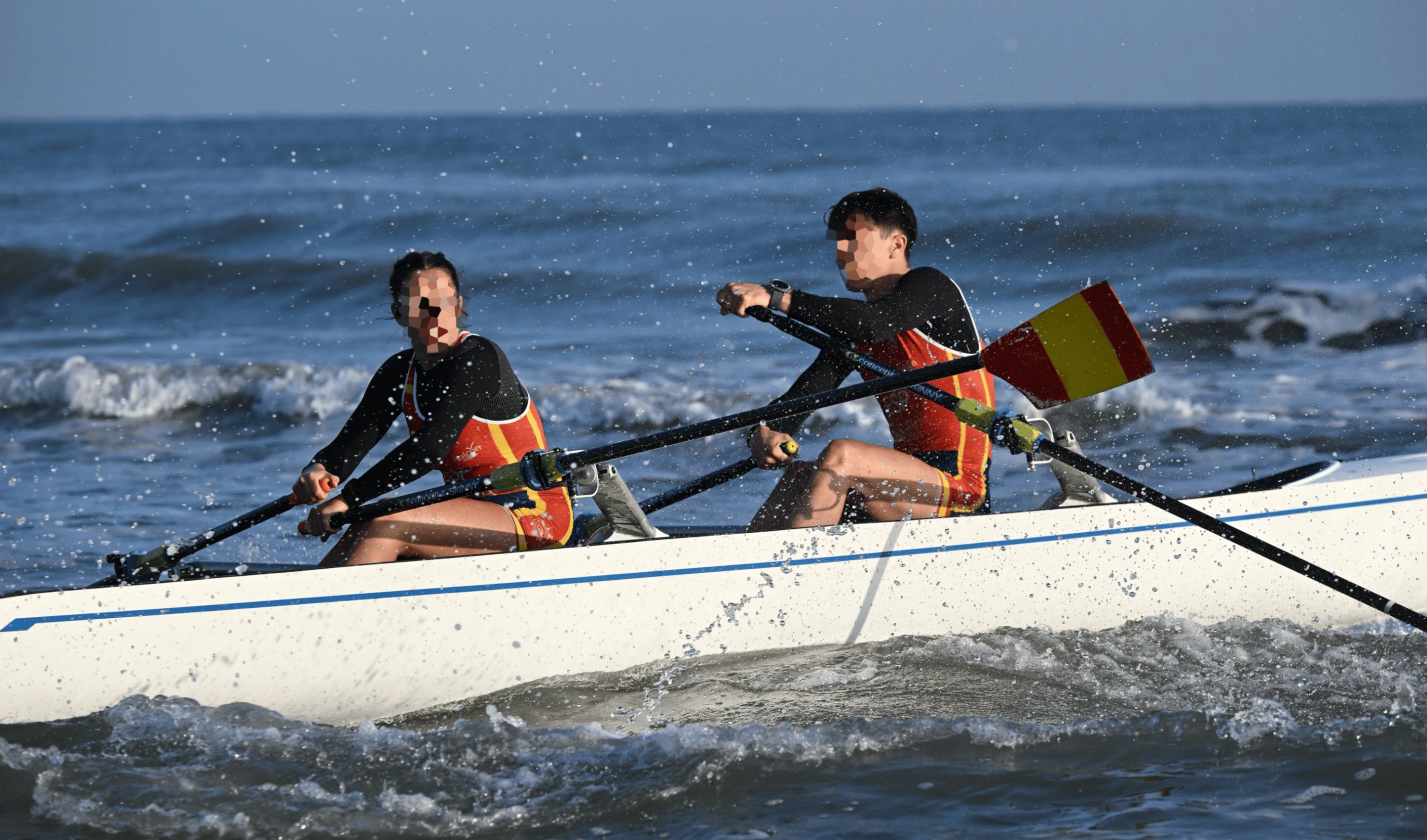
A coastal double in a wavy environment during the 2023 World Rowing Beach Sprint Finals in Barletta, Italy, highlighting an improper shoulder positioning, especially visible in the male rower

Shoulder injuries belong to the less common types of injuries in rowing and are mostly the result of imperfect technique and shoulder girdle positioning due to weakness or overuse in the muscles of the neck, shoulder, and anterior thorax [10,26]. An example of improper shoulder positioning can be seen in Figure 6, where the male rower pulls his shoulders up towards his head, resulting in increased tension in the muscles, such as the trapezius, increasing their vulnerability to overuse injuries. The resulting injuries are usually impingement or instability and pain in the rotator cuff, pectoralis muscles, and rhomboids due to overextended arms or generally increased tension in the shoulder girdle during the rowing stroke [10,27].

Coastal rowing beach sprint contains additional risks for shoulder injuries in contrast to classic Olympic rowing in the form of a difficult rowing environment, which poses a unique demand on the whole body, including the shoulders [3,12].

In addition to possibly existing technical imperfections, unexpected waves, sudden tipping of the boat, or strong forces and pressure on the oars during a turn in rough conditions can also lead to a potential increase in shoulder injuries compared to classic Olympic rowing [12,19,28]. Sprint rowing in general also leads to an increased risk for shoulder dislocations in younger rowers with shoulder instability, further increasing the potential risk for shoulder injuries in coastal rowing beach sprint [24,27].

Injuries of the Forearm and Wrist

**Figure 7:**
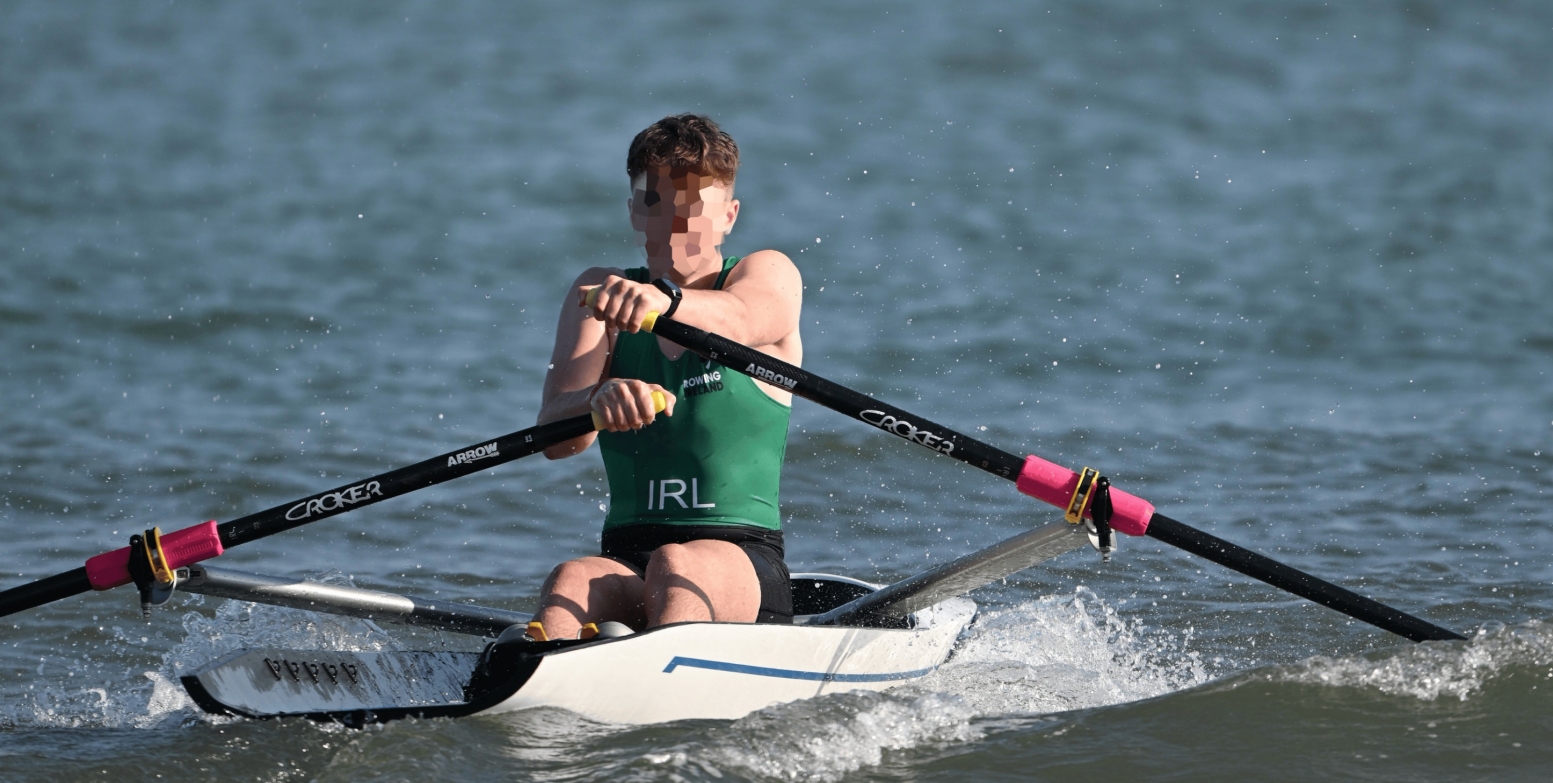
A single sculler during a race in the junior men’s single scull in 2023 at the World Rowing Beach Sprint Finals in Barletta, Italy His right hand is in a very stressful positioning, promoting the occurrence of injury.

Similar to the shoulder, most injuries regarding the upper extremity are caused by high training volume and bad rowing technique, mostly during the act of feathering or squaring the blade, where the wrist is placed in constant extension or flexion. Combined with a very tight grip, excessive motion of the boat, or the wrong hand position on the oars, these factors may lead to inflammation or swelling of the extending or flexing muscles of the wrist and forearm, lateral epicondylitis, De Quervain’s tenosynovitis, wrist extensor tenosynovitis, exertional compartment syndrome (ECS), and intersection syndrome [10,19,24]. Figure 7 shows a rower in the middle of a stroke, holding his right hand in a very stressful position during the rowing stroke. It is in full flexion while being slightly abducted towards the ulnar side.

The same can be said for kayaking and white-water rafting, where similar injuries occur as similar repetitive movements sometimes under stress from the environment in the form of waves or strong currents and swell are performed [19].

The risk for those and similar injuries could be increased in coastal rowing beach sprint compared to classic Olympic rowing, as the water is rougher and the course is shorter, promoting a higher stroke rate and therefore faster movement while under increased stress from bigger waves and surf [3].

Moreover, a lot of injuries of the lower arm in coastal rowing beach sprint, such as inflammations and tendosynovitis of the wrist extensors, appear one-sided (mostly left-sided) as a result of the sharp turn around the buoy (this has been observed and confirmed by the physiotherapist of the German Coastal Rowing Beach Sprint Team). This places the athlete's forearm muscles under new stress, not previously known from classic Olympic rowing competitions, as one oar is used to turn the boat, while the other arm holds the oar at one position to serve as an anker point around which the turn is made. In the coastal rowing beach sprint setting, the left arm is used to actively turn the boat around. Therefore, it is much more prone to before mentioned injuries as it must move the whole weight alone during the turn, presumably leading to far higher loads being placed on the muscles of a single arm, especially as coastal rowing beach sprint boats are heavier than those used in classic Olympic rowing.

Injuries of the Hands

Friction between the hands and oar frequently causes blisters and peeling skin on the hands of rowers [27]. Those very common minor lesions can lead to inflammation and pose a risk for infection with waterborne pathogens, especially in a very wavy environment where large amounts of water come in contact with blisters. Any other open wounds or injuries are a potential entryway for such infections as well [29,30].

**Figure 8:**
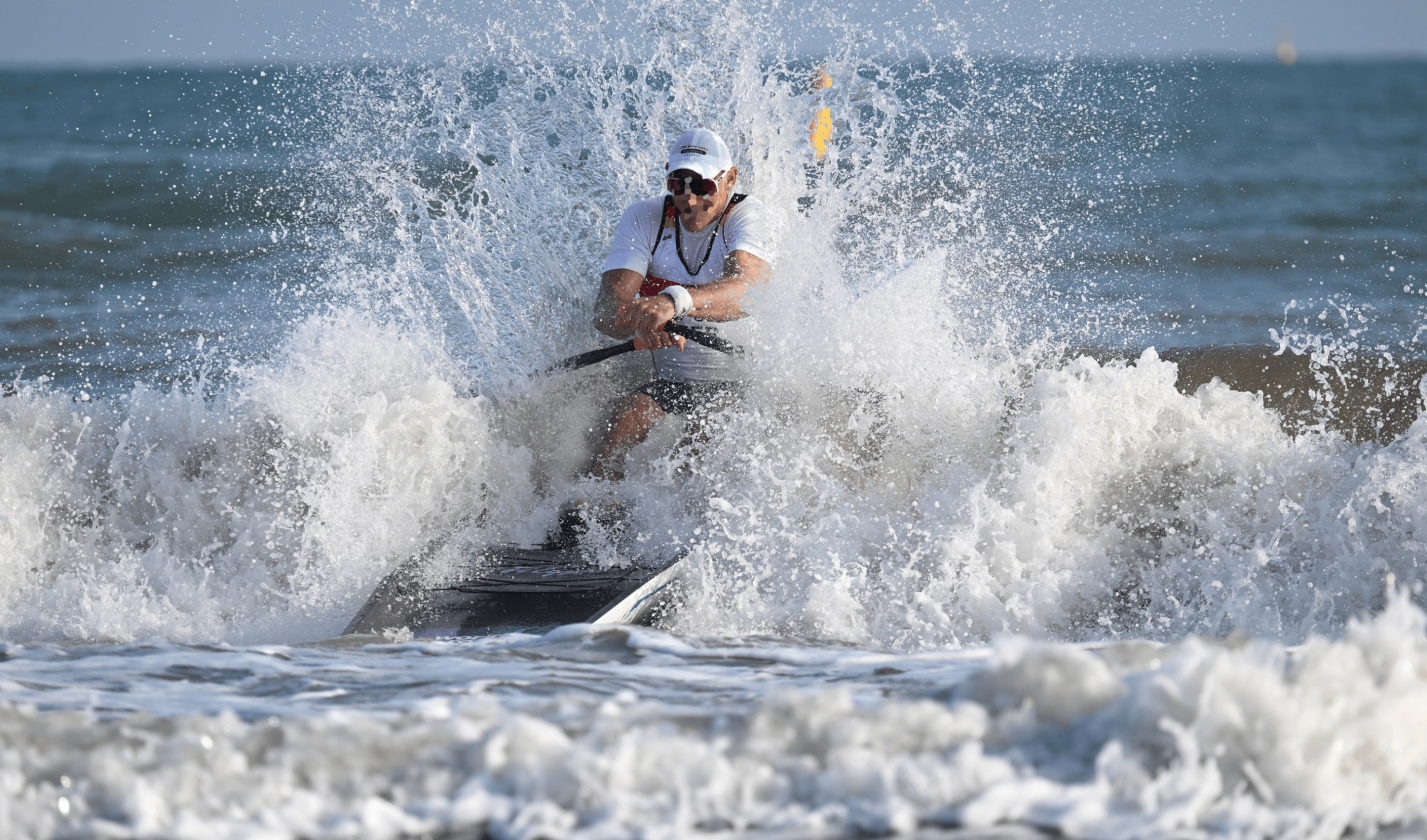
A wave hits a single sculler during the World Rowing Beach Sprint Finals in Barletta, Italy in 2023, highlighting how much water comes into contact with an athlete during racing

Although the above-mentioned infections are uncommon, they are still a risk to keep in mind, especially in coastal rowing beach sprint, as it includes a short run through the water towards the boat, as well as much more splashing water while rowing in the surf [3,29]. The splashing water during rowing can be seen in Figure 8.

Even though there have not been documented cases of an infection in coastal rowing beach sprint, the possibility remains and should be considered. Especially since ocean rowers frequently report infections [31].

Injuries of the Lower Extremity

**Figure 9:**
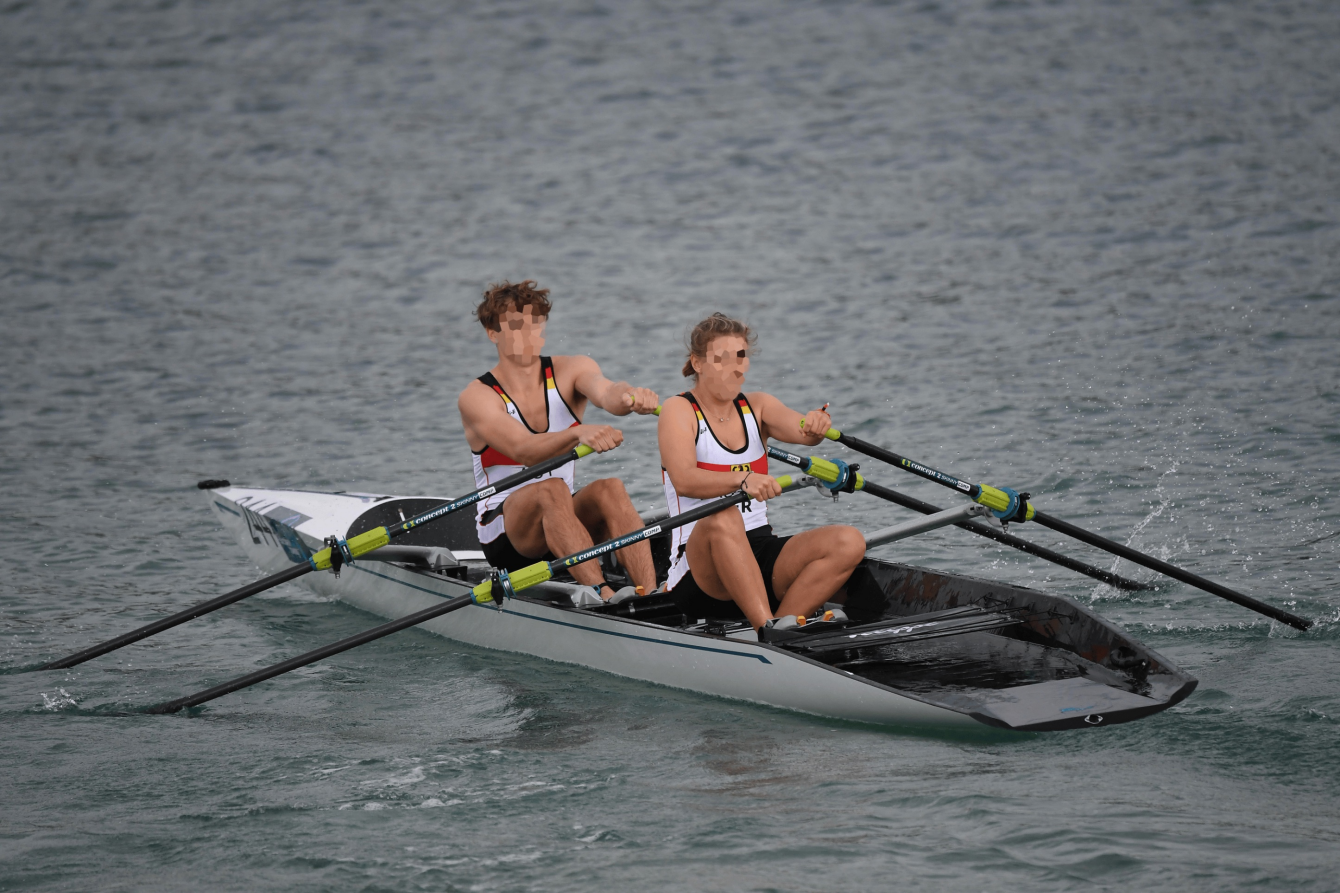
A German mixed double competing at the European Coastal Rowing Championships in 2022 in San Sebastian, Spain The woman on the right of the picture shows an imperfect knee position, which may result in knee injury if this positioning is kept during rowing for longer periods.

Contrary to popular belief, rowing is a sport relying heavily on leg strength to move the boat forward. As rowing is not a weight-bearing exercise, traumatic injuries are very unlikely to occur [10]. More common injuries of rowers regarding the lower extremity are irritations and inflammations of the retro patellar region, iliotibial or patellofemoral band structures [10,27]. Most of said injuries are the result of the nearly full range of motion in the knee during a complete rowing stroke, as well as possible technical imperfections, resulting in knee positions that place the bands over the lateral femoral condyle or the dorsal surface of the patella towards the femur under increased stress [10,27,32]. The rower on the right in Figure 9 shows a very imperfect positioning of her left knee, potentially resulting in extensive wear or inflammation if the technique is not adjusted.

In addition to injuries to the knee, there is also the possibility of injuries to the hip. The number of those injuries is increasing, most likely due to an improvement in diagnostic techniques and medical imaging [10]. Hip injuries are believed to be the result of anatomical deformities or variations combined with the extreme flexion in the hip during the catch of a rowing stroke [10,32].

All the above-mentioned injuries regarding the lower extremity will presumably also occur during coastal rowing beach sprint competitions or training due to the identical rowing motion. As mentioned before, the difficult water conditions could further increase the incidence of these injuries.

Another possible risk regarding the lower extremity could also be the infection of so-called “slide or track bites” over the calf muscle, similar to the infection of blisters on the hands [10]. These injuries are the result of continuous friction and resulting abrasion of the calf muscles against the slides on which the seat moves within the rowing boat [10].

**Figure 10:**
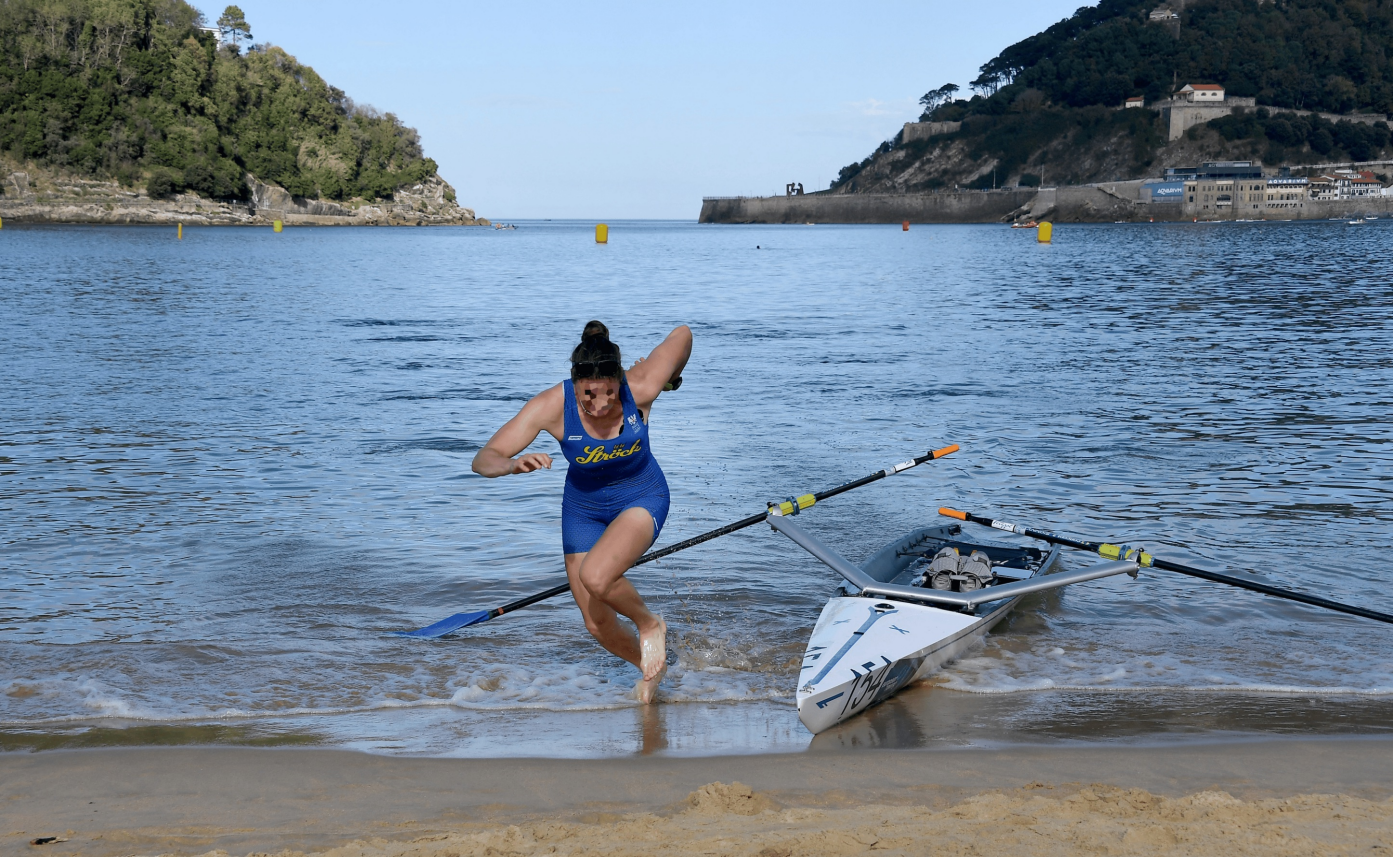
An Austrian single sculler sprinting towards the line in San Sebastian, Spain, during the European Coastal Championships in 2022

In addition to the rowing part, each coastal rowing beach sprint race includes two short sprints, which also contribute to an assumably different profile of potential injuries compared to classic Olympic rowing. The start of such a sprint is shown in Figure 10.

**Figure 11:**
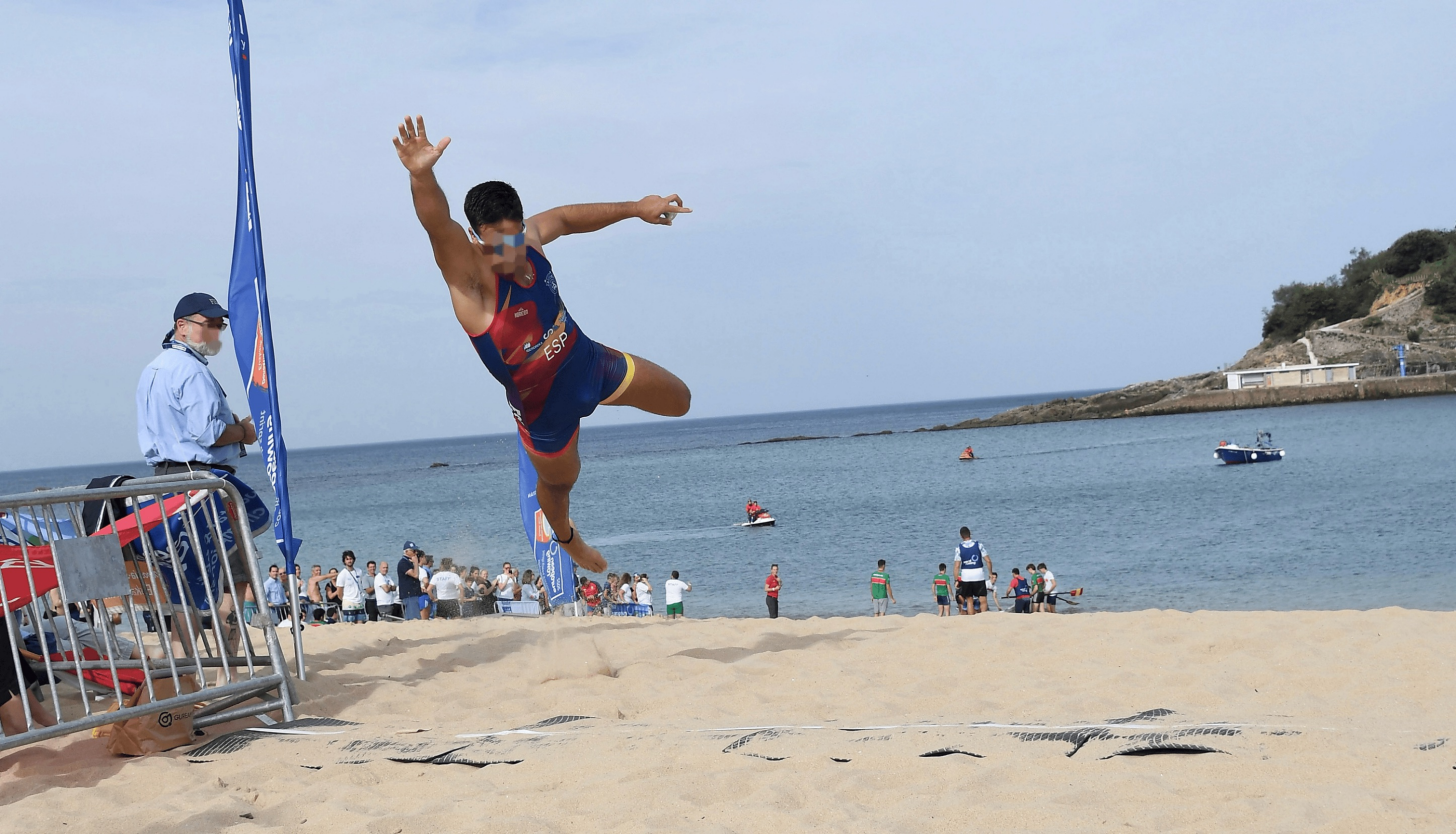
The final jump towards the line at the 2022 European Coastal Championships in San Sebastian, Spain

Even though the risk, especially that of ankle sprains, from running or exercising in the sand is significantly lower than that of comparable activities not performed on a sand surface the sudden changes of movement (running to rowing and back to running) comparable to triathlon competitions could lead to an increase in running injuries especially in the second sprint as studies show an increase in knee pain and fatigue in the knee after changing from one sport to another, specifically from cycling to running [33,34]. This change is quite similar to the change from rowing to running during coastal rowing beach sprint competitions. In addition, many injuries of the lower extremity in sports tend to happen during later parts of the competition, possibly because of fatigue [35]. The final jump towards the line, as seen in Figure 11, might also increase the amount of injuries, as sometimes increased risks such as big leaps towards the buzzer are taken to win the race. The affected regions would be tendons and muscles in the foot and lower leg. 

Beaches with pebbles or small stones are not as common as sand beaches, though some coastal rowing beach sprint competitions are still held at such locations, such as the 2024 World Rowing Beach Sprint Finals in Genoa. Stone or pebble beaches are most likely bringing along a greater risk for injuries during falls or ankle sprains, as sharp stones can harm the athletes and cause more damage than fine sand.

In addition to injuries linked to the rowing motion or running, the environment itself could also become a danger to beach sprint rowers. The swell and waves have been mentioned before, but small objects in the sand such as small stones or seashells can lead to cuts on the feet and possibly the hands during the sprint and while pressing the buzzer at the end of a race [36].

Environmental- and Material-Related Injuries

Apart from injuries related to the sport and its specific motion itself, coastal rowing beach sprint athletes also face the potential danger of injuries caused by their boats or the oars.

**Figure 12:**
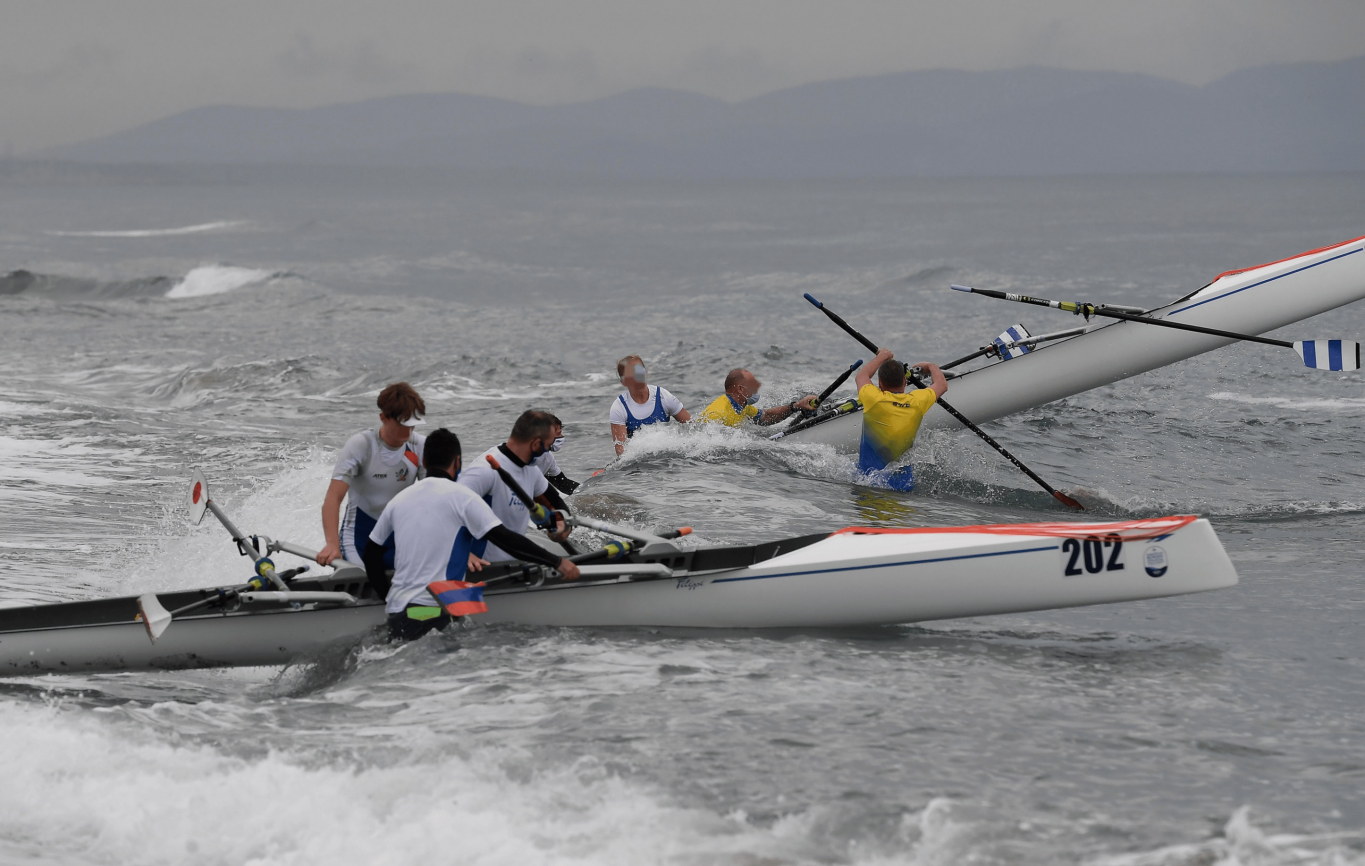
Volunteers try to keep the boats steady while battling the waves during the European Coastal Challenge 2022 in Donoratico, Italy

**Figure 13:**
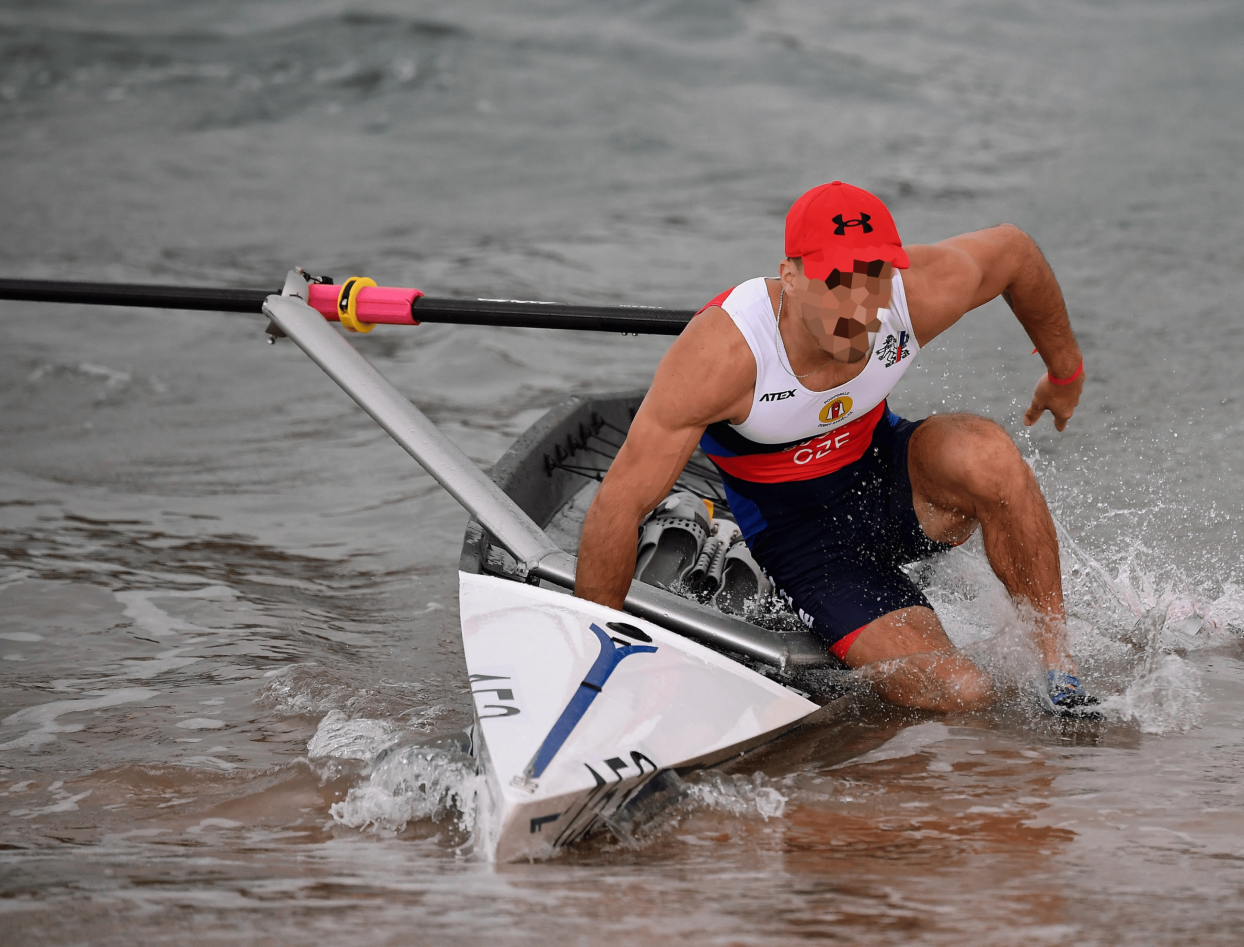
A Czech rower leaves his single to start the final sprint towards the line at the 2022 European Rowing Coastal Championships in San Sebastian, Spain

**Figure 14:**
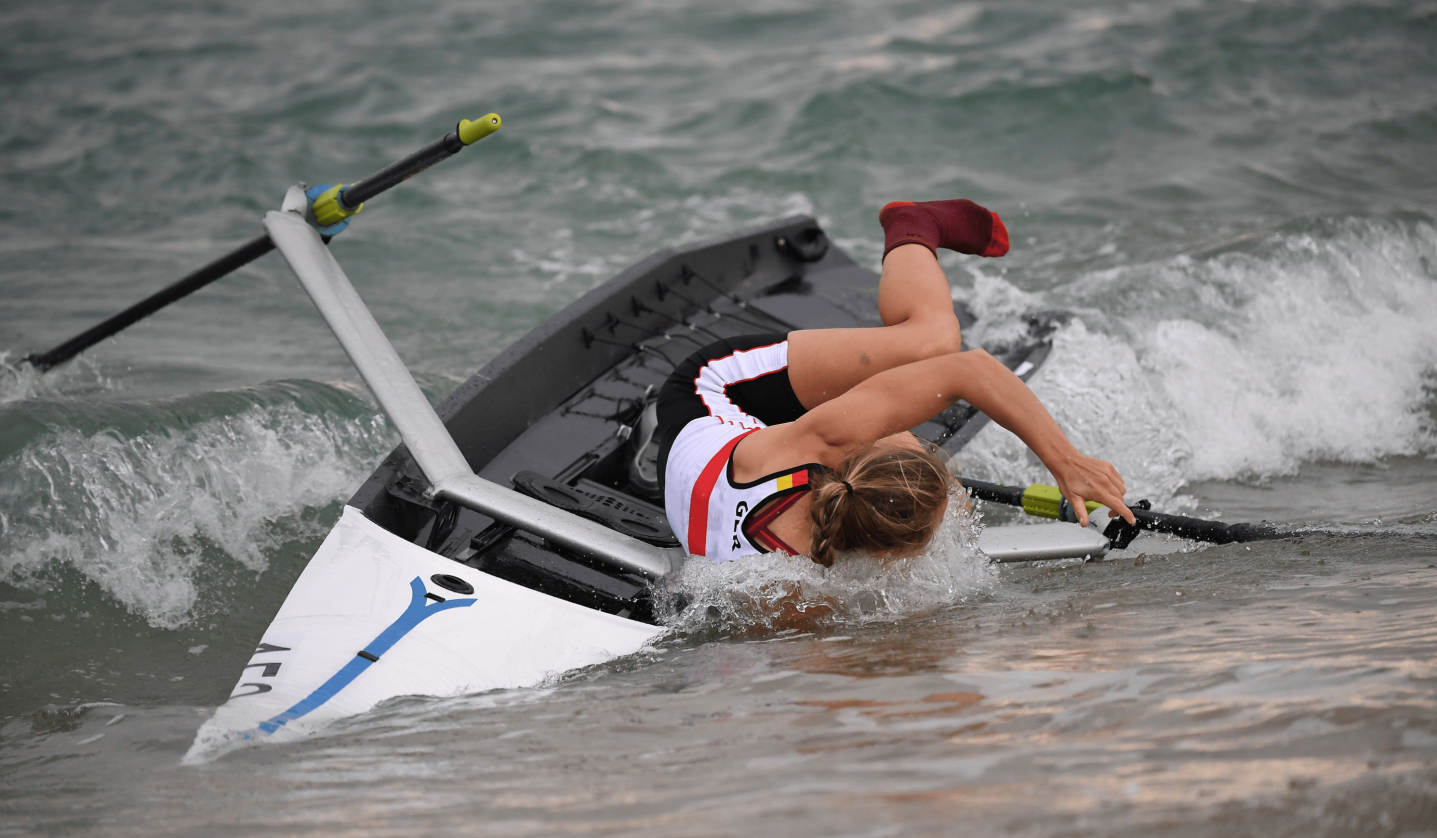
Single sculler falls from her boat at the 2022 European Rowing Coastal Championships in San Sebastian, Spain

The waves and climbing in and out of the boat in rough conditions and up to knee-deep water put rowers and boat handlers (helping hold the rowing boats steady before the rowers get in) at risk of being hit by the sports equipment. Getting in and out of the boat very quickly during races additionally poses the risk of twisting one’s ankle or wrist. Figure 12 shows the risks for boat handlers in a classic coastal rowing beach sprint environment, while Figures 13-14 depict athletes falling out of their boat while trying to start the final sprint towards the line along the beach.

Capsizing could also be much more likely in coastal rowing beach sprint compared to classic Olympic rowing due to the aforementioned water conditions, putting athletes at a presumably much bigger risk for drowning. In addition, rowers should consider the specific location and its weather.

Dehydration and exertional heat illness resulting from prolonged time and exhausting activities performed in the direct sun, combined with an insufficient fluid intake, can not only decrease physical and mental performance but also pose a potential health risk if extreme enough [37]. Moreover, sunburns are an immense risk factor for skin cancer and should also be considered as a potential injury, especially at competitions in regions with a high UV Index, as beaches do not offer a lot of shade for athletes and staff alike, thus making all people involved prone to beforementioned injuries [38].

Approach to improve medical care 

Classic Olympic rowing competitions always provide medical care in the form of an ambulance on standby, as well as a medical center at the venue and an on-water rescue team [39]. These measures have been adopted for coastal rowing beach sprint events as well [40]. Furthermore, the current weather, wave size, and wind are considered by the president of the jury, the organizing committee, and the race director, and racing and training can be stopped if danger is imminent [14]. In addition, the beaches are cleaned, and sharp stones, seashells, or washed-up wood and waste are removed to prevent injuries. As mentioned before, the lack of shade on the beach as a site of competition should be met with a sufficient amount of shelter to protect athletes and staff from the sun.

Due to a vastly different array of potential injuries, staff and medical personnel should also be prepared and equipped to treat possible injuries that are unlikely to occur in classic Olympic rowing.

Medical personnel should be ready to treat head, neck, and spine injuries caused by material, as well as unforeseen waves, and as a result of capsizing in shallow water. To ensure safe transport of injured athletes or boat handlers, spine boards, braces, and light stretchers should be available near the sprint area where athletes run and transition to and from their boats.

The short race distance of coastal rowing beach sprint and therefore very high stroke rate results in a potentially increased risk for shoulder dislocations, especially among the U19 athletes [24,27]. Responsible medical personnel should be familiar with reduction techniques and have bandages at hand.

Another injury potential that attending medical personnel and officials should be prepared for is ankle sprains and other leg injuries from sprinting and getting in and out of the boat (Table 2). For this instance, cooling packs and braces should be ready directly next to the sprint area.

**Table 2:** Potential injuries, mechanisms, and possible ways to improve medical care and reduce risks

Type of injury	Mechanism	Improvement of medical care and reduction of risks
Traumatic head or neck injuries	Hit by the rowing boat or the oars	Helmets and protective equipment, spine boards, braces and light stretchers should be available near the sprint area
Overuse injuries of muscles in the neck	Technical imperfections or wrong posture caused by waves etc.	Access to a physiotherapist/medical team, improve the technique
Irritation of the eyes	Splashing water (possibly contaminated with pathogens)	Eye protection
Lower back injuries (unspecific pain, inflammation, disc herniation)	Traumatic (waves, etc.), overuse	Access to a physiotherapist/medical team, controlled increase in training volume
Rib stress fracture	Exact injury mechanism unknown, muscular rib cage contractions, force transmission of the oar handle to the ribs or forces of the serratus anterior and external oblique muscles during the stroke	Access to a physiotherapist/medical team, controlled increase in training volume
Impingement, instability and pain in the rotator cuff, pectoralis muscles and rhomboids	Technical imperfections or wrong posture caused by waves, etc.	Access to a physiotherapist/medical team, improve the technique
Shoulder dislocations	Sprint rowing with a preexisting shoulder instability (especially younger rowers)	Be familiar with reduction techniques and have bandages at hand
Inflammation of the extending or flexing muscles of the wrist (often one sided), lateral epicondylitis, De Quervain’s tenosynovitis, wrist extensor tenosynovitis, exertional compartment syndrome (ECS) and intersection syndrome	Technical imperfections or wrong posture/grip caused by waves etc., harp and fast turning around a buoy at the end of the course	Access to a physiotherapist/medical team, improve the technique
Blisters and peeling skin on the hands and calf’s (potential infection)	Friction between the oar and the hand	Provide clean fresh water directly at the beach to give athletes the opportunity to clean these potential entry points
Inflammations of the retro patellar region, iliotibial or patellofemoral band structures	Almost full knee flexion during rowing, technical imperfections or wrong posture caused by waves etc.	Access to a physiotherapist/medical team, improve the technique
Hip injuries	Anatomical deformities or variations combined with the extreme flexion in the hip during the catch of a rowing stroke, technical imperfections or wrong posture caused by waves etc., splashing water or contact with the water during running	Access to a physiotherapist/medical team, improve the technique
Knee and ankle injuries (traumatic and overuse)	Sprinting in the sand, injuries caused by objects or stones lying in the sand	Beaches are cleaned, and sharp stones, seashells or washed-up wood and waste is removed, cooling packs and braces should be ready directly next to the sprint area, ankle bandages to reduce the chance of injury occurrence
Dehydration, exertional heat illness and sunburn	Lack of shade and insufficient fluid intake	Sufficient amount of shelter, provide clean fresh water directly at the beach
Traumatic injuries of the whole body	Hit by the rowing boat or the oars	Protective equipment such as helmets at least during high waves and stronger winds or wrist and ankle bandages to reduce the chance of injury occurrence

Especially in wavey conditions, during transitions into and out of the boat, falls, capsizing, or material-related incidents, rowers are prone to experience traumatic injuries to the upper and lower extremities, head, and trunk. Similar injuries can happen to boat handlers. A possible approach to reduce these kinds of injuries might be protective equipment, such as helmets at least during high waves and stronger winds, or wrist and ankle bandages to reduce the chance of injury occurrence.

Splashing water might infect blisters or other open wounds as well as the eyes. Even if it is difficult, organizers should provide clean, fresh water directly at the beach to give athletes the opportunity to clean these potential entry points for harmful microorganisms. In addition, this access to water reduces the risk of dehydration among everybody at the venue. Furthermore, athletes can protect possible entry sites by covering open wounds or wearing glasses to protect their eyes.

Many of the possible risks in coastal rowing beach sprint result in tight muscles, pain, or inflammation. In those cases, a physiotherapist at the location could help a lot to prevent a worsening of the issue. Due to high numbers of athletes attending coastal rowing beach sprint competitions, this measure is more suited to be organized by each individual team, especially as athletes can also get injured in training, so having access to a physiotherapist during training is recommended.

## Conclusions

Coastal rowing beach sprint will be part of the Olympic Games in Los Angeles, USA, in 2028. The demanding injury profile is a challenge not only for athletes and coaches but also for medical staff and officials. These new risk profiles require targeted preventive measures and medical care. In addition to proven and already implemented measures known from classic Olympic rowing, medical staff at the beach should be familiar with potential forms of injuries to provide sufficient care. Furthermore, specific infrastructure such as enough shade and access to fresh water and protective equipment for boat handlers in case of strong wind and waves can further improve the medical care in coastal rowing beach sprint.

Due to the limited number of research regarding coastal rowing beach sprint and rowing injuries in general, this narrative review could not use meta-analyses or systematic reviews as the only source but instead had to use the limited number of papers available on the topic. This affects the quality and underlines that further research is needed to improve the medical care for athletes in rowing even more.
